# Combined effects of lncRNA *MIR17HG* polymorphisms and lifestyle-related risk factors on the development and progression of tongue squamous cell carcinoma

**DOI:** 10.1042/BSR20260002

**Published:** 2026-05-14

**Authors:** Yi-Chieh Yang, Yi-Fang Ding, Chiao-Wen Lin, Yu-Fan Liu, Kuo-Hao Ho, Lun-Ching Chang, Shun-Fa Yang, Ming-Hsien Chien

**Affiliations:** 1 School of Oral Hygiene, College of Oral Medicine, Taipei Medical University, Taipei, Taiwan; 2 Department of Otolaryngology, Wan Fang Hospital, Taipei Medical University, Taipei, Taiwan; 3 Department of Otolaryngology, Chung Shan Medical University Hospital, Taichung, Taiwan; 4 Institute of Oral Sciences, Chung Shan Medical University, Taichung, Taiwan; 5 Department of Biomedical Sciences, College of Medicine Sciences and Technology, Chung Shan Medical University, Taichung, Taiwan; 6 Department of Biochemistry and Molecular Cell Biology, School of Medicine, College of Medicine, Taipei Medical University, Taipei, Taiwan; 7 Department of Mathematics and Statistics, Florida Atlantic University, Boca Raton, FL, U.S.A.; 8 Institute of Medicine, Chung Shan Medical University, Taichung, Taiwan; 9 Department of Medical Research, Chung Shan Medical University Hospital, Taichung, Taiwan; 10 Graduate Institute of Clinical Medicine, College of Medicine, Taipei Medical University, Taipei, Taiwan; 11 Pulmonary Research Center, Wan Fang Hospital, Taipei Medical University, Taipei, Taiwan; 12 Traditional Herbal Medicine Research Center, Taipei Medical University Hospital, Taipei, Taiwan; 13 TMU Research Center of Cancer Translational Medicine, Taipei Medical University, Taipei, Taiwan; 14 School of Medical Laboratory Science and Biotechnology and PhD Program in Medical Biotechnology, College of Medical Science and Technology, Taipei Medical University, Taipei, Taiwan

**Keywords:** Asian male population, lifestyle-related risk, MIR17HG, oral squamous cell carcinoma, progression, single-nucleotide polymorphism

## Abstract

Oral squamous cell carcinoma is the most common malignancy of the oral cavity, with tongue squamous cell carcinoma (TSCC) being particularly prevalent worldwide. Evidence suggests that the long noncoding RNA, miRNA 17–92a-1 cluster host gene (*MIR17HG*), plays dual roles in tumor development. However, its clinical relevance and genetic variation in TSCC remain unclear. Analysis of data from TCGA revealed that *MIR17HG* expression is significantly up-regulated in head and neck squamous cell carcinoma, particularly in male and Asian populations. The present study examined the association between *MIR17HG* single-nucleotide polymorphisms (SNPs) and TSCC progression in Taiwanese men. Four tagging SNPs (rs1428, rs7318578, rs17735387, and rs75267932) were genotyped through a TaqMan allelic discrimination assay. The results revealed that rs75267932 G allele carriers had a significantly elevated risk of developing large tumors in the dominant genetic model. This association was more prominent in patients exposed to one of the environmental carcinogens, including betel quid, cigarette smoke, or alcohol. Smokers or drinkers carrying the rs75267932 variant were also likely to present with advanced disease. When patients with TSCC were stratified by exposure to all three lifestyle-related risk factors, the effect of rs75267932 on disease progression became the strongest. Conversely, non-betel-quid chewers or nonsmokers carrying the variant had a reduced risk of high-grade tumors. Overall, the effect of rs75267932 on TSCC progression was most pronounced in individuals exposed to lifestyle-related risk factors. These findings unveil gene–environment interactions in TSCC and highlight *MIR17HG* polymorphisms as potential biomarkers for risk assessment and disease monitoring in Asian men.

## Introduction

Oral squamous cell carcinoma (OSCC) is among the six most common types of cancer worldwide. OSCC predominantly affects the tongue, resulting in tongue squamous cell carcinoma (TSCC). Traditionally, OSCC is more prevalent among men than among women [1]. Its etiology is complex and involves multiple risk factors, such as persistent alcohol consumption, tobacco use, and human papillomavirus infection [2,3]. Betel nut chewing is another predominant risk factor for OSCC in Taiwan [4]. Although the pathogenesis of OSCC remains unclear, evidence suggests that this disease results from the combined effects of multiple risk factors and genetic and epigenetic alterations. Despite substantial advancements in treatment modalities, including surgery, postoperative radiotherapy or chemotherapy, and immunotherapy, the 5-year survival rate of patients with OSCC remains unsatisfactory [5,6], likely because of delayed diagnosis, which allows for tumor metastasis. More than 60% of all OSCC cases are identified at advanced stages [7]. Therefore, reliable predictive factors and therapeutic targets for OSCC must be urgently identified.

MicroRNAs (miRNAs) are small, single-stranded, highly conserved noncoding RNAs consisting of 17–25 nucleotides. When multiple miRNA genes are located in close proximity on a chromosome, they form an miRNA gene cluster, which is typically regulated by the host gene promoter [7]. The human miRNA 17–92a-1 cluster host gene (*MIR17HG*), located on chromosome 13q31.3 within the third intron of *c13orf25*, spans approximately 800 nucleotides and encodes 6 miRNAs, namely miR-17, miR-18a, miR-19a, miR-20a, miR-19b-1, and miR-92a-1, as a long noncoding RNA (lncRNA). Functional research has implicated *MIR17HG* in cell survival, proliferation, differentiation, and angiogenesis [8]. This gene plays dual roles as both an oncogene and a tumor suppressor across different types of cancer. Regarding its oncogenic role, *MIR17HG* promotes metastasis in gastric cancer by regulating Wnt/β-catenin signaling [9]. It also functions as a competing endogenous RNA to up-regulate HK1 by sponging miR-138-5p, thereby enhancing glycolysis and promoting colorectal cancer invasion and liver metastasis [10]. Conversely, *MIR17HG* suppresses breast cancer cell proliferation and migration by serving as a competing endogenous RNA that targets *FAM135A* by sponging miR-454-3p [11]. Moreover, *MIR17HG* inhibits the motility of non-small-cell lung cancer by up-regulating miR-142-3p to down-regulate Bach-1 [12]. These context-dependent oncogenic and tumor-suppressive roles highlight the complex functions of *MIR17HG* in cancer.

Single-nucleotide polymorphisms (SNPs) are widely distributed within lncRNA genes, where they directly or indirectly modulate lncRNA expression through multiple mechanisms, thereby influencing cancer development and progression [13]. Several *MIR17HG* SNPs have been associated with cancer risk, disease progression, or patient survival across different malignancies. For example, rs7318578 has been associated with an increased glioma risk, whereas rs17735387 has been associated with favorable prognosis [14]. Research has suggested that the risk of liver cancer is higher in individuals carrying the CC genotype of rs7318578 than in those carrying the AA genotype [15]. Furthermore, rs7336610, rs7318578, rs17735387, and rs1428 have been strongly associated with colorectal cancer risk in men [16]. To date, the effects of *MIR17HG* polymorphisms on the development and progression of OSCC, particularly TSCC in Asian populations, remain largely unclear. In this case–control study, we examined the associations of *MIR17HG* SNPs with OSCC risk and clinical features in a Taiwanese cohort. We also explored the associations of *MIR17HG* SNPs with OSCC progression risk in patients stratified by lifestyle-related risk factors.

## Materials and methods

### Study cohort

The present study included 382 men with TSCC who sought treatment at Chung Shan Medical University Hospital (Taichung, Taiwan). All participants provided informed consent and information on their exposure to lifestyle-related risk factors for cancer, such as alcohol consumption, cigarette smoking, and betel nut chewing. In addition, 1189 anonymized healthy controls were randomly selected from the Taiwan Biobank Project. None of these individuals had a history of cancer at any site. Individuals with oral precancerous conditions, such as oral submucosal fibrosis, erythroplakia, leukoplakia, or verrucous hyperplasia, were excluded from the control group. The following clinical data were obtained from the medical records of patients with TSCC: tumor–node–metastasis clinical stage, primary tumor size, lymph node involvement, distant metastasis, and histologic grade. The study protocol was approved by the Ethics Committee of Chung Shan Medical University Hospital (approval number: CS1-21151).

### Genomic DNA extraction and *MIR17HG* SNP selection

Genomic DNA extraction was performed according to our previous study [17,18]. Briefly, whole blood samples were collected from all participants into tubes containing ethylenediaminetetraacetic acid and centrifuged at 3000 rpm to isolate the buffy coat. Genomic DNA was subsequently extracted from blood leukocytes by using a QIAamp DNA Mini Kit (Qiagen, Valencia, CA, U.S.A.) following the manufacturer’s protocol. DNA purity and concentration were assessed using a NanoDrop-2000 spectrophotometer (Thermo Fisher Scientific, Waltham, MA, U.S.A.). The samples were stored at −20°C before genotyping. Four *MIR17HG* SNPs (rs75267932, rs17735387, rs7318578, and rs1428) were selected on the basis of data from the National Center for Biotechnology Information dbSNP database [19], using the following criteria: minor allele frequency >5% and pairwise linkage disequilibrium value <0.8. Notably, these four SNPs were selected because of their established associations with cancer risk or progression in the Han Chinese population [14–16].

### Genotyping of *MIR17HG* SNPs

Genotyping of *MIR17HG* SNPs was performed using an ABI StepOnePlus™ RT-PCR System (Applied Biosystems, Foster City, CA, U.S.A.) with individual TaqMan SNP probes: rs75267932 (assay ID: C_101836836_10), rs17735387 (assay ID: C__33435792_20), rs7318578 (assay ID: C__30460419_10), and rs1428 (assay ID: C___1409672_10). The genotyping results were analyzed using SDS software version 3.0 (Applied Biosystems). The detailed procedures have been previously described [20].

### Data collection from bioinformatics analyses

Data from the University of Alabama at Birmingham Cancer (UALCAN) online database (http://ualcan.path.uab.edu, accessed on 1 December 2025) [21] were analyzed to evaluate the expression of *MIR17HG* and *ZBTB38* in normal and primary tumor tissues and identify the associations of *ZBTB38* expression with clinicopathological characteristics (e.g., tumor grade) in patients with head and neck squamous cell carcinoma (HNSCC) retrieved from The Cancer Genome Atlas (TCGA) dataset. In addition, UALCAN data were used to assess subgroup differences across demographic variables, such as age, sex, and race. Moreover, potential targets of miR-4684-5p in TSCC cells were identified using data from the miRDB database (http://mirdb.org/).

### Assessment of lncRNA–miRNA binding stability through thermodynamic modeling

To evaluate knockdown potential, the thermodynamic interface between the lncRNA and miRNA was analyzed *in silico*. BiBiServ2-RNAhybrid [22] was used to predict RNA–RNA interactions, hybridization energy values, and duplex structures. With “human” selected as the target source and output limited to one hit per target, the probabilities of base pairing and corresponding interaction energies were calculated. Structural linearity and the hydrogen-bond index were assessed to evaluate the stability of predicted hybridization.

### Statistical analysis

Demographic variables were compared between cancer-free individuals (healthy controls) and patients with TSCC by using the Mann–Whitney *U* test or Fisher’s exact test. Odds ratios (ORs) with corresponding 95% confidence intervals (CIs) were calculated using logistic regression to identify the associations of genotype frequencies with TSCC risk and clinicopathological characteristics. Betel quid chewing, cigarette smoking, and alcohol consumption were included as covariates in adjusted models. All statistical analyses were conducted using SAS software version 9.1 (SAS Institute, San Francisco, CA, U.S.A.).

## Results

### *MIR17HG* expression is elevated in HNSCC, particularly among Asian male patients

**Figure 1:**
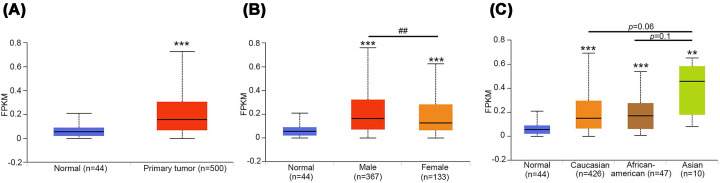
Clinical significance of *MIR17HG* in patients with HNSCC Data from the UALCAN database were analyzed. (**A**) *MIR17HG* expression levels in normal and tumor tissues. *MIR17HG* expression levels in tumor tissues from patients stratified by sex (**B**) and race (**C**). Significance: **P <0.01 and ***P <0.001, compared with the normal group; ^##^*P* <0.01, compared with female patients.

To determine the expression levels of *MIR17HG* and their clinical significance in oral cancer, cases of HNSCC were analyzed using data from the UALCAN database. According to RNA sequencing data from TCGA, the levels of *MIR17HG* transcripts were significantly higher in tumor tissues than in normal tissues (*P* <0.001; Figure 1A). This finding highlighted an oncogenic role of *MIR17HG* in HNSCC tumorigenesis. Subgroup analyses were conducted by stratifying patients with HNSCC by multiple demographic variables, including sex and race. The results indicated that the levels of *MIR17HG* were significantly higher in male than in female patients (Figure 1B), suggesting sex-related regulatory mechanisms or hormonal effects. *MIR17HG* expression was markedly higher in Asian patients than in Caucasian and African-American patients (Figure 1C), indicating that differences in ethnic or genetic background contribute to dysregulated expression.

### General characteristics of the study cohort

Given that *MIR17HG* contributes to HNSCC tumorigenesis in Asian men, we recruited 382 Taiwanese men with TSCC and 1189 healthy controls. Table 1 presents the baseline characteristics of the study cohort. No significant differences were observed between the case and control groups in terms of age distribution, indicating appropriate comparability. However, the frequencies of alcohol consumption, betel quid chewing, and cigarette smoking were significantly higher in the case group than in the control group (*P* <0.001). These lifestyle patterns are consistent with those noted in Asian patients with OSCC [23,24], confirming that these behaviors represent major environmental and lifestyle-related risk factors for TSCC carcinogenesis. Regarding clinical features, the majority of patients with TSCC exhibited no sign of lymph node metastasis (60.7%) or distant metastasis (99.5%), reflecting a predominance of localized or regionally confined disease at diagnosis. Approximately 88.7% of all tumors were moderately or poorly differentiated, suggesting aggressive histopathological characteristics within the cohort.

**Table 1:** Demographic characteristics of the study cohort

Variable	Control group (*N* = 1189), *n* (%)	Patient group (*N* = 382), *n* (%)	*P*
Age (years)			
<60	775 (65.2)	244 (63.9)	0.642
≥60	414 (34.8)	138 (36.1)	
Betel quid chewing			
No	991 (83.3)	130 (34.0)	
Yes	198 (16.7)	252 (66.0)	<0.001*
Cigarette smoking			
No	558 (46.9)	88 (23.0)	
Yes	631 (53.1)	294 (77.0)	<0.001*
Alcohol consumption			
No	952 (80.1)	237 (62.0)	
Yes	237 (19.9)	145 (38.0)	<0.001*
Stage			
I + II	−	168 (44.0)	
III + IV	−	214 (56.0)	
Tumor T status			
T1 + T2	−	195 (51.0)	
T3 + T4	−	187 (49.0)	
Lymph node status			
N0	−	232 (60.7%)	
N1 + N2 + N3	−	150 (39.3%)	
Metastasis			
M0	−	380 (99.5)	
M1	−	2 (0.5)	
Cell differentiation			
Well differentiated	−	43 (11.3)	
Moderately or poorly differentiated	−	339 (88.7)	

Significance: *P <0.05.

### Associations of *MIR17HG* SNPs with TSCC risk

To identify the associations of the selected *MIR17HG* SNPs—two exonic SNPs (rs75267932 and rs1428) and two intronic SNPs (rs7318578 and rs17735387)—with TSCC in our Taiwanese cohort, we calculated the adjusted ORs with corresponding 95% CIs by using multiple logistic regression models adjusted for potential confounders (betel nut chewing, cigarette smoking, and alcohol consumption). We identified the most common genotypes as homozygous A/A for rs7318578 and rs75267932, homozygous G/G for rs17735387, and heterozygous A/C for rs1428 (Table 2). Next, we investigated the associations between *MIR17HG* SNPs and TSCC risk in codominant and dominant genetic models. In contrast with the frequencies of corresponding wild-type (WT) genotypes, those of *MIR17HG* SNPs exhibited no significant differences between the case and control groups.

**Table 2:** Associations between the risk of tongue cancer and the frequencies of *MIR17HG* polymorphisms

Variable	Control group (*N* = 1,189), *n* (%)	Patient group (*N* = 382), *n* (%)	aOR (95% CI)	*P*
**rs1428**				
AA	314 (26.4)	104 (27.2)	1.000 (reference)	
AC	626 (52.6)	183 (47.9)	0.837 (0.611–1.145)	0.265
CC	249 (21.0)	95 (24.9)	1.169 (0.808–1.691)	0.408
AC + CC	875 (73.6)	278 (72.8)	0.928 (0.691–1.247)	0.621
**rs7318578**				
AA	670 (56.3)	214 (56.0)	1.000 (reference)	
AC	448 (37.7)	134 (35.1)	0.938 (0.709–1.241)	0.653
CC	71 (6.0)	34 (8.9)	1.504 (0.913–2.479)	0.109
AC + CC	519 (43.7)	168 (44.0)	1.016 (0.781–1.323)	0.905
**rs17735387**				
GG	738 (62.1)	234 (61.3)	1.000 (reference)	
GA	404 (34.0)	132 (34.6)	1.036 (0.784–1.369)	0.803
AA	47 (3.9)	16 (4.1)	1.154 (0.593–2.246)	0.674
GA + AA	451 (37.9)	148 (38.7)	1.048 (0.801–1.371)	0.733
**rs75267932**				
AA	911 (76.6)	295 (77.2)	1.000 (reference)	
AG	262 (22.0)	82 (21.5)	1.220 (0.886–1.679)	0.223
GG	16 (1.3)	5 (1.3)	0.967 (0.301–3.109)	0.955
AG + GG	278 (23.4)	87 (22.8)	1.204 (0.881–1.645)	0.245

Adjusted odds ratios with corresponding 95% CIs were calculated using multiple logistic regression models adjusted for betel quid chewing, cigarette smoking, and alcohol consumption.

### Associations of *MIR17HG* SNPs with the clinicopathological characteristics of patients with TSCC

We examined whether *MIR17HG* SNPs were associated with the clinicopathological characteristics of patients with TSCC—for example, primary tumor size, clinical stage, metastatic status, and histopathologic grade. Our results indicated that patients with TSCC carrying a minor allele (AG or GG) of rs75267932 had a higher risk of developing larger tumors (>T2; OR = 2.122, 95% CI = 1.297–3.473, *P* = 0.002) compared with those carrying the WT homozygous genotype (AA). This finding suggests that rs75267932 plays a role in promoting tumor growth or enhancing tumor aggressiveness in TSCC. However, the other three *MIR17HG* SNPs (rs1428, rs7318578, and rs17735387) exhibited no significant associations with the clinicopathological variables analyzed in the present study (Tables 3 and 4). Thus, rs75267932 emerged as the primary SNP associated with TSCC progression, whereas the remaining SNPs exerted limited or no effects on the clinical behavior of the disease.

**Table 3:** Clinical conditions and rs1428 and rs7318578 frequencies in patients with tongue cancer

Variable	rs1428 (*N* = 382)				rs7318578 (*N* = 382)			
	AA (*N* = 104), *n* (%)	AC + CC (*N* = 278), *n* (%)	OR (95% CI)	*P*	AA (*N* = 214), *n* (%)	AC + CC (*N* = 168), *n* (%)	OR (95% CI)	*P*
**Clinical stage**								
I + II	49 (47.1)	119 (42.8)	1.000 (reference)	0.450	94 (43.9)	74 (44.0)	1.000 (reference)	0.981
III + IV	55 (52.9)	159 (57.2)	1.190 (0.757–1.871)		120 (56.1)	94 (56.0)	0.995 (0.662–1.495)	
**Tumor size**								
≤T2	51 (49.0)	144 (51.8)	1.000 (reference)	0.631	110 (51.4)	85 (50.6)	1.000 (reference)	0.876
>T2	53 (51.0)	134 (48.2)	0.895 (0.571–1.405)		104 (48.6)	83 (49.4)	1.033 (0.689–1.547)	
**Lymph node metastasis**								
No	65 (62.5)	167 (60.1)	1.000 (reference)	0.665	131 (61.2)	101 (60.1)	1.000 (reference)	0.828
Yes	39 (37.5)	111 (39.9)	1.108 (0.697–1.762)		83 (38.8)	67 (39.9)	1.047 (0.692–1.583)	
**Cell differentiation**								
Well differentiated	10 (9.6)	33 (11.9)	1.000 (reference)	0.535	24 (11.2)	19 (11.3)	1.000 (reference)	0.977
Moderately or poorly differentiated	94 (90.4)	245 (88.1)	0.790 (0.374–1.666)		190 (88.8)	149 (88.7)	0.991 (0.523–1.877)	

ORs with corresponding 95% CIs were calculated using logistic regression models.

**Table 4:** Clinical conditions and rs17735387 and rs75267932 frequencies in patients with tongue cancer

Variable	rs17735387 (*N* = 382)				rs75267932 (*N* = 382)			
	GG (*N* = 234), *n* (%)	GA + AA (*N* = 148), *n* (%)	OR (95% CI)	*P*	AA (*N* = 295), *n* (%)	AG + GG (*N* = 87), *n* (%)	OR (95% CI)	*P*
**Clinical stage**								
I + II	108 (46.2)	60 (40.5)	1.000 (reference)	0.282	136 (46.1)	32 (36.8)	1.000 (reference)	0.124
III + IV	126 (53.8)	88 (59.5)	1.257 (0.829–1.907)		159 (53.9)	55 (63.2)	1.470 (0.899–2.405)	
**Tumor size**								
≤T2	120 (51.3)	75 (50.7)	1.000 (reference)	0.908	163 (55.3)	32 (36.8)	1.000 (reference)	0.002*
>T2	114 (48.7)	73 (49.3)	1.025 (0.679–1.547)		132 (44.7)	55 (63.2)	**2.122 (1.297–3.473)**	
**Lymph node metastasis**								
No	145 (62.0)	87 (58.8)	1.000 (reference)	0.535	180 (61.0)	52 (59.8)	1.000 (reference)	0.834
Yes	89 (38.0)	61 (41.2)	1.142 (0.750–1.739)		115 (39.0)	35 (40.2)	1.054 (0.647–1.717)	
**Cell differentiation**								
Well differentiated	23 (9.8)	20 (13.5)	1.000 (reference)	0.267	30 (10.2)	13 (14.9)	1.000 (reference)	0.216
Moderately or poorly differentiated	211 (90.2)	128 (86.5)	0.698 (0.369–1.321)		265 (89.8)	74 (85.1)	0.644 (0.320–1.298)	

ORs with corresponding 95% CIs were calculated using logistic regression models.

Significance: *P <0.05.

### Stratified analysis of the associations between rs75267932 SNPs and the clinicopathological characteristics of patients with TSCC

Chewing betel quid has been identified as one of the most potent risk factors for OSCC within East Asian populations [25]. Evidence from Taiwan, where betel quid consumption is endemic, indicates an association between betel quid chewing and unfavorable clinical outcomes in OSCC [26]. In the present study, we stratified patients with TSCC into betel quid chewers and nonchewers to determine whether *MIR17HG* SNPs are differentially associated with clinicopathological characteristics in these exposure-defined populations. Our results indicated that the association between the carriage of at least one rs75267932 G allele and the development of advanced tumors (>T2) was stronger in betel quid chewers than in the overall TSCC cohort (Table 5). Conversely, among nonchewers, carriers of at least one mutant allele of rs75267932 exhibited a significantly reduced likelihood of presenting with high-grade TSCC (moderately or poorly differentiated tumors; Table 5). We further analyzed the modifying effects of tobacco use and alcohol consumption on the associations between *MIR17HG* SNPs and TSCC progression. Among patients who smoked or consumed alcohol, those carrying at least one rs75267932 G allele exhibited a higher risk of developing larger tumors than did WT carriers (Tables 6 and 7). These exposure groups also exhibited an increased likelihood of presenting with advanced disease (stage III or IV; Tables 6 and 7). Among patients without a history of smoking, carriers of the rs75267932 variant allele had a reduced risk of developing high-grade tumors (Table 6). When patients with TSCC were stratified by cumulative exposure to the three lifestyle-related risk factors, the effect of rs75267932 on tumor progression became more evident, underscoring potential synergistic interactions between genetic susceptibility and environmental risk factors (Table 8).

**Table 5:** Clinical conditions and rs75267932 frequencies in patients with tongue cancer stratified by betel quid chewing

Variable	Nonchewers (*N* = 130)				Chewers (*N* = 252)			
	AA (*N* = 98), *n* (%)	AG + GG (*N* = 32), *n* (%)	OR (95% CI)	*P*	AA (*N* = 197), *n* (%)	AG + GG (*N* = 55), *n* (%)	OR (95% CI)	*P*
**Clinical stage**								
I + II	43 (43.9)	13 (40.6)	1.000 (reference)	0.747	93 (47.2)	19 (34.5)	1.000 (reference)	0.095
III + IV	55 (56.1)	19 (59.4)	1.143 (0.508–2.570)		104 (52.8)	36 (65.5)	1.694 (0.909–3.157)	
**Tumor size**								
≤T2	54 (55.1)	13 (40.6)	1.000 (reference)	0.155	109 (55.3)	19 (34.5)	1.000 (reference)	0.006*
>T2	44 (44.9)	19 (59.4)	1.794 (0.798–4.032)		88 (44.7)	36 (65.5)	**2.347 (1.259–4.375)**	
**Lymph node metastasis**								
No	56 (57.1)	17 (53.1)	1.000 (reference)	0.691	124 (62.9)	35 (63.6)	1.000 (reference)	0.925
Yes	42 (42.9)	15 (46.9)	1.176 (0.528–2.622)		73 (37.1)	20 (36.4)	0.971 (0.522–1.806)	
**Cell differentiation**								
Well differentiated	7 (7.1)	7 (21.9)	1.000 (reference)	0.020*	23 (11.7)	6 (10.9)	1.000 (reference)	0.875
Moderately or poorly differentiated	91 (92.9)	25 (78.1)	**0.275 (0.088–0.857)**		174 (88.3)	49 (89.1)	1.080 (0.416–2.799)	

ORs with corresponding 95% CIs were calculated using logistic regression models.

Significance: *P <0.05.

**Table 6:** Clinical conditions and rs75267932 frequencies in patients with tongue cancer stratified by cigarette smoking

Variable	Nonsmokers (*N* = 88)				Smokers (*N* = 294)			
	AA (*N* = 69), *n* (%)	AG + GG (*N* = 19), *n* (%)	OR (95% CI)	*P*	AA (*N* = 226), *n* (%)	AG + GG (*N* = 68), *n* (%)	OR (95% CI)	*P*
**Clinical stage**								
I + II	29 (42.0)	10 (52.6)	1.000 (reference)	0.410	107 (47.3)	22 (34.5)	1.000 (reference)	0.029*
III + IV	40 (58.0)	9 (47.4)	0.653 (0.235–1.809)		119 (52.7)	46 (67.6)	**1.880 (1.062–3.329)**	
**Tumor size**								
≤T2	33 (47.8)	10 (52.6)	1.000 (reference)	0.711	130 (57.5)	22 (32.4)	1.000 (reference)	<0.001*
>T2	36 (52.2)	9 (47.4)	0.825 (0.298–2.281)		96 (42.5)	46 (67.6)	**2.831 (1.597–5.019)**	
**Lymph node metastasis**								
No	38 (55.1)	11 (57.9)	1.000 (reference)	0.826	142 (62.8)	41 (60.3)	1.000 (reference)	0.705
Yes	31 (44.9)	8 (42.1)	0.891 (0.319–2.489)		84 (37.2)	27 (39.7)	1.113 (0.639–1.940)	
**Cell differentiation**								
Well differentiated	4 (5.8)	5 (26.3)	1.000 (reference)	0.009*	26 (11.5)	8 (11.8)	1.000 (reference)	0.953
Moderately or poorly differentiated	65 (94.2)	14 (73.7)	**0.172 (0.0 0.724)**		200 (88.5)	60 (88.2)	0.975 (0.420–2.266)	

ORs with corresponding 95% CIs were calculated using logistic regression models.

Significance: *P <0.05.

**Table 7:** Clinical conditions and rs75267932 frequencies in patients with tongue cancer stratified by alcohol consumption

Variable	Nondrinkers (*N* = 237)				Drinkers (*N* = 145)			
	AA (*N* = 185), *n* (%)	AG + GG (*N* = 52), *n* (%)	OR (95% CI)	*P*	AA (*N* = 110), *n* (%)	AG + GG (*N* = 35), *n* (%)	OR (95% CI)	*P* value
**Clinical stage**								
I + II	82 (44.3)	23 (44.2)	1.000 (reference)	0.990	54 (49.1)	9 (25.7)	1.000 (reference)	0.015*
III + IV	103 (55.7)	29 (55.8)	1.004 (0.540–1.865)		56 (50.9)	26 (74.3)	**2.786 (1.196–6.486)**	
**Tumor size**								
≤T2	97 (52.4)	23 (44.2)	1.000 (reference)	0.296	66 (60.0)	9 (25.7)	1.000 (reference)	<0.001*
>T2	88 (47.6)	29 (55.8)	1.390 (0.749–2.580)		44 (40.0)	26 (74.3)	**4.333 (1.855–10.124)**	
**Lymph node metastasis**								
No	113 (61.1)	32 (61.5)	1.000 (reference)	0.952	67 (60.9)	20 (57.1)	1.000 (reference)	0.692
Yes	72 (38.9)	20 (38.5)	0.981 (0.521–1.846)		43 (39.1)	15 (42.9)	1.169 (0.540–2.527)	
**Cell differentiation**								
Well differentiated	18 (9.7)	9 (17.3)	1.000 (reference)	0.129	12 (10.9)	4 (11.4)	1.000 (reference)	0.932
Moderately or poorly differentiated	167 (90.3)	43 (82.7)	0.515 (0.216–1.226)		98 (89.1)	31 (88.6)	0.949 (0.285–3.155)	

ORs with corresponding 95% CIs were calculated using logistic regression models.

Significance: *P <0.05.

**Table 8:** Clinical conditions and rs75267932 frequencies in patients with tongue cancer stratified by cigarette smoking, alcohol consumption, and betel quid chewing

Variable	Without tobacco, alcohol, and betel quid exposure (*N* = 64)				With tobacco, alcohol, and betel quid exposure (*N* = 115)			
	AA (*N* = 49), *n* (%)	AG + GG (*N* = 15), *n* (%)	OR (95% CI)	*P*	AA (*N* = 88), *n* (%)	AG + GG (*N* = 27), *n* (%)	OR (95% CI)	*P*
**Clinical stage**								
I + II	22 (44.9)	8 (53.3)	1.000 (reference)	0.567	48 (54.5)	7 (25.9)	1.000 (reference)	0.009*
III + IV	27 (55.1)	7 (46.7)	0.713 (0.223–2.275)		40 (45.5)	20 (74.1)	**3.429 (1.316–8.933)**	
**Tumor size**								
≤T2	24 (49.0)	8 (55.3)	1.000 (reference)	0.768	58 (65.9)	7 (25.9)	1.000 (reference)	<0.001*
>T2	25 (51.0)	7 (46.7)	0.840 (0.264–2.676)		30 (34.1)	20 (74.1)	**5.524 (2.100–14.528)**	
**Lymph node metastasis**								
No	30 (61.2)	9 (60.0)	1.000 (reference)	0.932	58 (65.9)	16 (59.3)	1.000 (reference)	0.528
Yes	19 (38.8)	6 (40.0)	1.053 (0.323–3.433)		30 (34.1)	11 (40.7)	1.329 (0.548–3.221)	
**Cell differentiation**								
Well differentiated	3 (6.1)	5 (33.3)	1.000 (reference)	0.005*	12 (13.6)	3 (11.1)	1.000 (reference)	0.733
Moderately or poorly differentiated	46 (93.9)	10 (66.7)	**0.130 (0.027–0.637)**		76 (86.4)	24 (88.9)	1.263 (0.329–4.852)	

ORs with corresponding 95% CIs were calculated using logistic regression models.

Significance: *P <0.05.

## Discussion

Evidence suggests that lncRNAs play key roles in cancer development. Research has identified multiple cancer-associated lncRNAs, including those involved in OSCC [27,28]. *MIR17HG*, a lncRNA located on human chromosome 13q31, modulates the development and progression of various types of human cancer by regulating tumor growth and apoptosis. For example, overexpression of *MIR17HG* down-regulates a proapoptotic gene during the development of lung cancer [29]. *MIR17HG* encodes the polycistronic miR-17-92 cluster, which contains six miRNAs (miR-17, miR-18a, miR-19a, miR-20a, miR-19b-1, and miR-92a-1). Each member of this cluster plays a unique role across different types of cancer. For instance, miR-18a and miR-92a promote cell proliferation, cell migration, and cell-cycle progression in lung cancer by targeting Sprouty 4 [30]. Conversely, miR-18a suppresses the proliferation of bladder cancer cells by down-regulating *DICER1* [31]. The miR-17-92 cluster plays a complex role in OSCC, with individual members exhibiting divergent biological activities. Notably, miR-92a and miR-19a promote the proliferation and motility of OCSS cells by targeting forkhead box P1 and G protein-coupled receptor kinase 6, respectively [32,33]. By contrast, miR-17/20a and miR-18a suppress tumor motility by targeting integrin β8 and hypoxia-inducible factor-1α, respectively [34,35]. Collectively, these findings suggest that the miR-17-92 cluster plays both oncogenic and tumor-suppressive roles in OSCC, depending on the specific miRNA and cellular context. *MIR17HG* may influence the aberrant expression of the miR-17-92 cluster and has been reported to play a distinct role in human papilloma virus–related HNSCC [36].

Genetic variants can influence gene expression or alter gene structure, thereby contributing to cancer progression. *MIR17HG* polymorphisms are strongly associated with the development of several types of cancer [14–16]. Our study revealed that carriers of the rs75267932 G allele exhibited a significantly increased risk of developing larger tumors in the dominant model (AG + GG). By contrast, Xu et al. [37] reported that rs7336610 and rs1428, but not rs75267932, were associated with an increased HNSCC risk in the Chinese Han population. Although both studies involved Asian populations, our cohort exclusively included patients with TSCC, whereas the cohort analyzed by Xu et al. [37] predominantly included patients with thyroid or nasopharyngeal squamous cell carcinoma. Interstudy differences in tumor types may explain the observed discrepancies.

Betel quid chewing, smoking, alcohol consumption, and combinations of these habits have been implicated in the pathogenesis of OSCC. In the present study, we analyzed the combined effects of *MIR17HG* SNPs and these lifestyle-related risk factors on TSCC progression. Our findings revealed that the association between rs75267932 and an increased risk of developing larger tumors was stronger in patients with TSCC who chewed betel quid, smoked cigarettes, or consumed alcohol than in the overall TSCC cohort. Patients with TSCC who smoked or consumed alcohol and carried the rs75267932 variant were more likely to present with advanced disease. However, among patients without a history of betel quid chewing or smoking, carriers of the rs75267932 variant had a reduced risk of developing high-grade tumors. Notably, both the tumor-promoting and the protective effects of this SNP became more pronounced when patients with TSCC were stratified by combined exposure or nonexposure to lifestyle-related risk factors. Together, these findings suggest that specific *MIR17HG* polymorphisms influence the clinical progression of TSCC, particularly in individuals with certain lifestyle exposure patterns. These habits have been associated with substantial epigenetic changes, such as methylation of DNA, modification of histone proteins, and dysregulation of noncoding RNAs that drive cancer progression. For example, the DLK1-MEG3 imprinting locus contains the large miR-379/656 cluster, which serves as a tumor suppressor [38] and is epigenetically silenced in Taiwanese patients with OSCC who chew betel nuts [39]. Notably, miR-487b is a tumor suppressor that is silenced through epigenetic mechanisms during tobacco-induced pulmonary carcinogenesis [40]. We propose that these lifestyle-related risk factors epigenetically regulate the miR-17-92 cluster to promote OSCC progression; however, this hypothesis requires further investigation.

**Figure 2:**
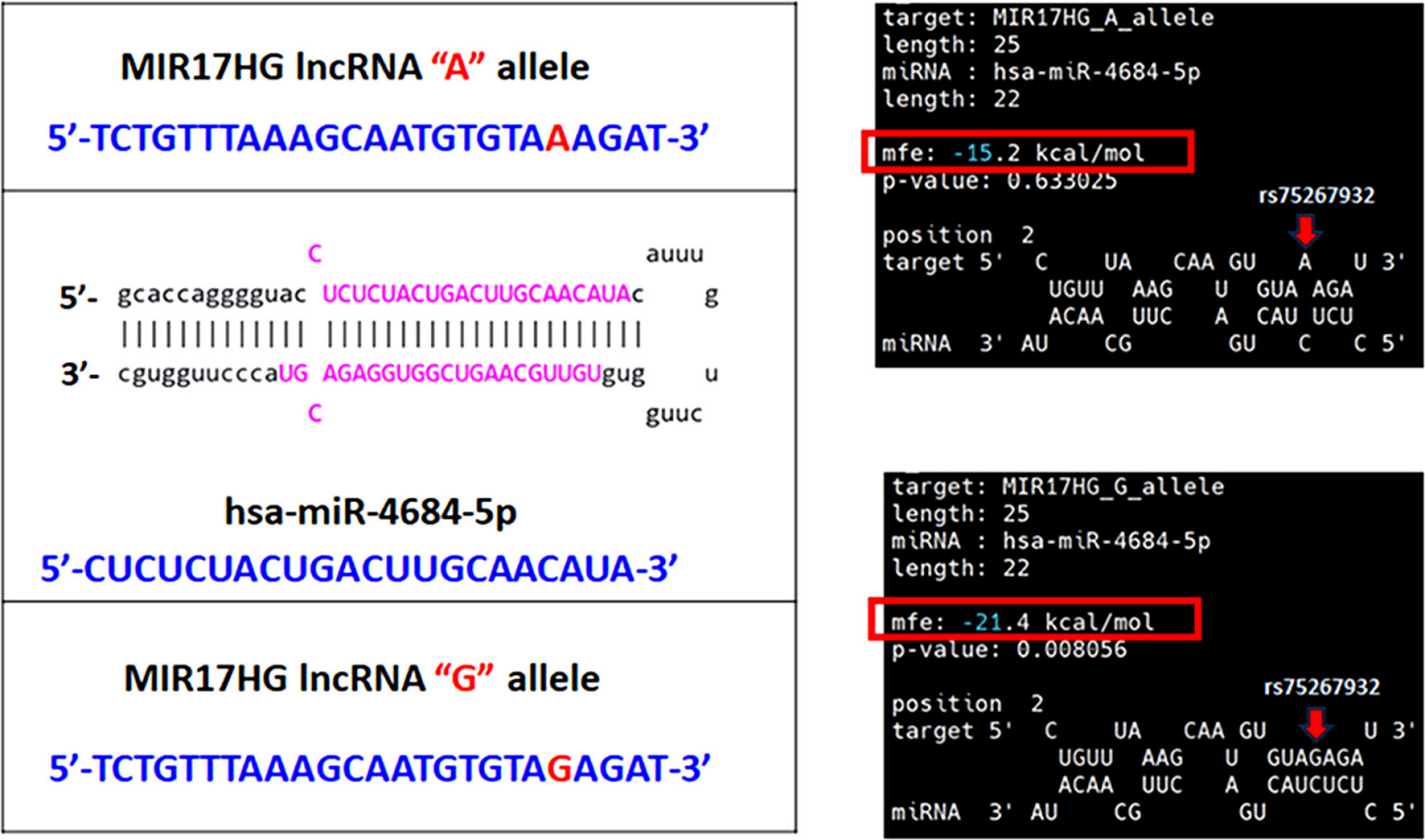
Predicted hybridization between the lncRNA *MIR17HG* and interacting miRNAs Left panel: predicted structures from the hybridization of *MIR17HG* containing the rs75267932 A or G allele with miR-4684-5p. Right panel: the rs75267932 G allele significantly stabilized the binding of miR-4684-5p, reducing the minimum free energy by 40.8%, as indicated by analyses conducted using BiBiServ2-RNAhybrid. The location of rs75267932 is indicated by red arrows.

**Figure 3:**
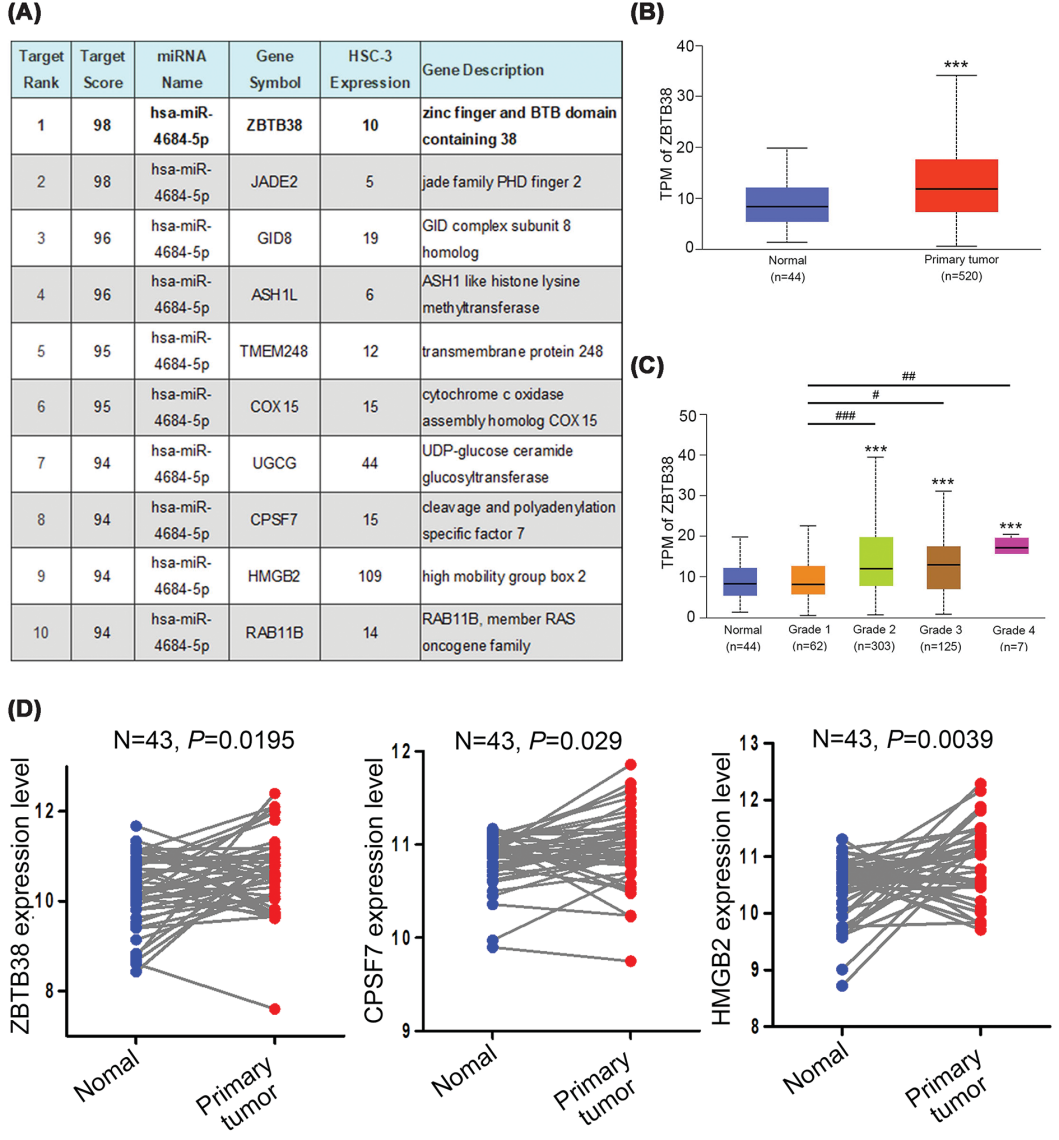
The predicted target genes of miR-4684-5p in HSC-3 TSCC cells and the clinical significance of the top-ranked target in patients with HNSCC (**A**) Analysis of miRDB target expression data revealed the top 10 targets of miR-4684-5p with expression levels of ≥5 in HSC-3 cells. (**B**) Data from the UALCAN database were used to compare *ZBTB38* expression levels in normal and HNSCC tissues. (**C**) *ZBTB38* expression levels in tumor tissues from patients with HNSCC stratified by tumor grade. Significance: ***P <0.001, compared with normal tissues; ^#^*P* <0.05, ^##^*P* <0.01, and ^###^*P* <0.001, compared with grade I HNSCC tissues. (**D**) Transcript levels of *ZBTB38, CPSF7*, and *HMGB2* in 43 paired normal and primary tumor tissue samples. TSCC, tongue squamous cell carcinoma; HNSCC, head and neck squamous cell carcinoma.

A growing body of evidence suggests that *MIR17HG* functions as a molecular sponge that binds specific miRNAs and subsequently up-regulates the expression of their target genes to modulate cancer progression. For example, *MIR17HG* promotes the metastasis of colorectal cancer by sponging miR-138-5p and up-regulating the expression of its target gene *HK1* [10]. *MIR17HG* also sponges miR-21, thereby increasing *PTEN* expression and regulating chemoresistance in acute myeloid leukemia cells [41]. Numerous SNPs influence miRNA–lncRNA interactions [42]. In our study, the potential interactions between candidate miRNAs and rs75267932 were predicted using BiBiServ2-RNAhybrid [22]. We observed that the hybridization energy was lower for the interaction between the rs75267932 A allele and miR-4684-5p than for that between the rs75267932 G allele and miR-4684-5p (Figure 2). Therefore, the G allele enhances the binding affinity of *MIR17HG* for miR-4684-5p (*P* = 0.0081). To date, limited information is available on the association between miR-4684-5p and cancer. Only one study published on Research Square (https://www.researchsquare.com/article/rs-31503/v1) reported that the lncRNA *FAM83H-AS1* promotes tumorigenesis in esophageal squamous cell carcinoma by sponging miR-4684-5p and subsequently up-regulating the expression of its target gene *ZBTB38*. The miRNA target prediction database miRDB [43] ranks *ZBTB38* as the top target of miR-4684-5p in HSC-3 TSCC cells (Figure 3A). In addition to HSC-3 cells, the miRDB database also identifies *ZBTB38* as the top predicted target of miR-4684-5p in other TSCC cell lines, including CAL-27, SCC-9, and SCC-15 (Supplementary Figure S1). An analysis of data from TCGA-HNSCC cohort revealed significantly higher *ZBTB38* transcript levels in tumor tissues than in normal tissues (Figure 3B) and in higher-grade tumors than in lower-grade ones (Figure 3C), highlighting a potential oncogenic role of *ZBTB38* in HNSCC. In addition to *ZBTB38, CPSF7*, and *HMGB2* expression levels were also significantly elevated in TCGA-paired normal and primary HNSCC samples (Figure 3D). Taken together, these findings suggest that *MIR17HG* functions as a competing endogenous RNA to sponge miR-4684-5p, thereby up-regulating the oncogenic targets, including *ZBTB38, CPSF7*, and *HMGB2*, and accelerating OSCC development. The rs75267932 SNP in *MIR17HG* may modulate the interaction between *MIR17HG* and miR-4684-5p. The detailed regulatory circuitry underlying these interactions needs to be further evaluated.

Several limitations should be acknowledged. First, the present study was not conducted as a double-blind randomized controlled trial; therefore, residual bias may remain. Second, all TSCC cases included in the SNP analysis were derived from a Taiwanese population, whereas the clinicopathological correlations of *MIR17HG* expression were evaluated using the TCGA-HNSCC cohort, which predominantly comprises non-Asian individuals. Therefore, further validation in Taiwanese cohorts is warranted. Additionally, the lack of long-term follow-up data limited our ability to evaluate survival outcomes associated with *MIR17HG* polymorphisms. Finally, established risk factors for oral cancer progression, including periodontal disease and chronic *Porphyromonas gingivalis* infection, were not adjusted for in the multivariable analysis.

To the best of our knowledge, the present study is the first to demonstrate the distinct allelic effects of the *MIR17HG* SNP rs75267932 and their contribution to the pathogenesis of TSCC in a male Taiwanese population. The combined effects of the rs75267932 genotype and lifestyle-related risk factors (betel nut chewing, cigarette smoking, and alcohol consumption) further underscore a causal role of this SNP in TSCC development. These findings highlight a potential synergistic interaction between genetic susceptibility and environmental exposure. We further observed that the rs75267932 G allele may enhance the binding affinity of miR-4684-5p to *MIR17HG*, thereby accelerating OSCC progression through the up-regulation of *ZBTB38*. Nevertheless, these findings should be validated in future studies.

## Supplementary Material

Supplementary Figure S1

## Data Availability

The data supporting the current findings are included within the article. Further inquiries can be directed to the corresponding authors.

## Abbreviations

CIs: confidence intervals
HNSCC: head and neck squamous cell carcinoma
lncRNA: long noncoding RNA
miRNA: microRNA
MIR17HG: miRNA 17–92a-1 cluster host gene
ORs: odds ratios
OSCC: oral squamous cell carcinoma
SNP: single-nucleotide polymorphism
TCGA: The Cancer Genome Atlas
TSCC: tongue squamous cell carcinoma
WT: wild type
