# Supplementary material for: Combined effects of lncRNA MIR17HG polymorphisms and lifestyle-related risk factors on the development and progression of tongue squamous cell carcinoma
Source: Biosci Rep. 2026 May 14;46(5):BSR20260002. doi: 10.1042/BSR20260002 (PMC13181356; doi:10.1042/BSR20260002)
Supplement: Supplementary Figure S1 [file BSR-2026-0002_supp.pdf]

Supplemental Information

Title:

Combined effects of lncRNA MIR17HG polymorphisms and lifestyle-related risk factors on the development and progression of tongue squamous cell carcinoma

Yi-Chieh Yang, Yi-Fang Ding, Chiao-Wen Lin, Yu-Fan Liu, Kuo-Hao Ho, Lun-Ching Chang, Shun-Fa Yang, and Ming-Hsien Chien

Correspondence to: Dr. Shun-Fa Yang (E-mail: ysf@csmu.edu.tw) and Dr. Ming-Hsien Chien (E-mail: d002089002@tmu.edu.tw)

Figure Legends

TSCC: CAL-27, SCC-9, SCC-15

Target Rank	Target Score	miRNA Name	Gene Symbol	CAL 27 Expression	Gene Description
1	98	hsa-miR-4684-5p	ZBTB38	11	zinc finger and BTB domain containing 38
2	98	hsa-miR-4684-5p	JADE2	42	jade family PHD finger 2
3	96	hsa-miR-4684-5p	CXXC5	7	CXXC finger protein 5
4	96	hsa-miR-4684-5p	ASH1L	5	ASH1 like histone lysine methyltransferase
5	96	hsa-miR-4684-5p	GID8	23	GID complex subunit 8 homolog
6	95	hsa-miR-4684-5p	TMEM248	17	transmembrane protein 248
7	95	hsa-miR-4684-5p	COX15	18	cytochrome c oxidase assembly homolog COX15
8	94	hsa-miR-4684-5p	UGCG	55	UDP-glucose ceramide glucosyltransferase
9	94	hsa-miR-4684-5p	RAB11B	42	RAB11B, member RAS oncogene family
10	94	hsa-miR-4684-5p	HMGB2	47	high mobility group box 2

Target Rank	Target Score	miRNA Name	Gene Symbol	SCC-9 Expression	Gene Description
1	98	hsa-miR-4684-5p	ZBTB38	9	zinc finger and BTB domain containing 38
2	96	hsa-miR-4684-5p	GID8	15	GID complex subunit 8 homolog
3	96	hsa-miR-4684-5p	CXXC5	14	CXXC finger protein 5
4	96	hsa-miR-4684-5p	ASH1L	7	ASH1 like histone lysine methyltransferase
5	95	hsa-miR-4684-5p	COX15	9	cytochrome c oxidase assembly homolog COX15
6	95	hsa-miR-4684-5p	TMEM248	22	transmembrane protein 248
7	94	hsa-miR-4684-5p	RAB11B	27	RAB11B, member RAS oncogene family
8	94	hsa-miR-4684-5p	HMGB2	60	high mobility group box 2
9	94	hsa-miR-4684-5p	UGCG	19	UDP-glucose ceramide glucosyltransferase
10	94	hsa-miR-4684-5p	CPSF7	26	cleavage and polyadenylation specific factor 7

Target Rank	Target Score	miRNA Name	Gene Symbol	SCC-15 Expression	Gene Description
1	98	hsa-miR-4684-5p	ZBTB38	6	zinc finger and BTB domain containing 38
2	98	hsa-miR-4684-5p	JADE2	5	jade family PHD finger 2
3	96	hsa-miR-4684-5p	ASH1L	5	ASH1 like histone lysine methyltransferase
4	96	hsa-miR-4684-5p	GID8	21	GID complex subunit 8 homolog
5	96	hsa-miR-4684-5p	CXXC5	9	CXXC finger protein 5
6	95	hsa-miR-4684-5p	TMEM248	20	transmembrane protein 248
7	95	hsa-miR-4684-5p	COX15	10	cytochrome c oxidase assembly homolog COX15
8	94	hsa-miR-4684-5p	HMGB2	42	high mobility group box 2
9	94	hsa-miR-4684-5p	RAB11B	26	RAB11B, member RAS oncogene family
10	94	hsa-miR-4684-5p	CPSF7	11	cleavage and polyadenylation specific factor 7

Figure S1. The predicted target genes of miR-4684-5p in several tongue squamous cell carcinoma (TSCC) cell lines. Analysis of the miRDB database (https://mirdb.org/) was performed to predict potential target genes of miR-4684-5p. Based on the target score and expression profiles, the top 10 predicted targets with expression levels ≥5 were selected for further evaluation in CAL-27, SCC-9, and SCC-15 TSCC cell lines.
